# Behavioural Repeatability and Behavioural Syndrome in the Dung Beetle *Copris umbilicatus* (Coleoptera, Scarabaeidae)

**DOI:** 10.3390/insects14060529

**Published:** 2023-06-06

**Authors:** Gianluca Natta, Alex Laini, Angela Roggero, Fabrizio Fabbriciani, Antonio Rolando, Claudia Palestrini

**Affiliations:** 1 Department of Life Science and Systems Biology, University of Turin, Via Accademia Albertina 13, 10123 Torino, Italy; gianluca.natta@unito.it (G.N.); alex.laini@unito.it (A.L.); antonio.rolando@unito.it (A.R.); claudia.palestrini@unito.it (C.P.); 2 Independent Researcher, Via Alfredo Chiti 9, 51100 Pistoia, Italy; f.fabbriciani@libero.it; 3 National Biodiversity Future Center (NBFC), 90133 Palermo, Italy

**Keywords:** distress signal, locomotor activity, thanatosis, behavioural traits, sex, body size

## Abstract

**Simple Summary:**

Not only vertebrates but also invertebrates can display personalities and behavioural syndromes. Here, three multiple behaviours (activity, thanatosis and distress call emission) were investigated in *Copris umbilicatus*. Moderate to excellent levels of repeatability were found in all behavioural traits considered. Results suggest the existence of a behavioural syndrome involving thanatosis and activity, with ‘bolder’ individuals exhibiting shorter thanatosis and higher locomotor activity, in contrast with ‘fearful’ individuals which display longer thanatosis and poor locomotor activity. Noticeable differences between individuals, which were not due to differences in sex or body size, could be attributable to differences in personality among individuals.

**Abstract:**

Although personality studies have primarily focused on vertebrates, the evidence showing invertebrates to be capable of displaying personalities has been steadily growing in recent years. In this study, we investigated the behavioural repeatability (repetition of a behaviour over time) and behavioural syndromes (a set of correlated behaviours) in *Copris umbilicatus*, which is a dung beetle species showing complex sub-social behaviour. We analysed three behaviours (activity, thanatosis and distress call emission) by measuring seven distinct behavioural traits (i.e., three activity-, one thanatosis- and three distress call-related traits). We found moderate to high levels of individual repeatability in all behavioural traits considered. The duration of thanatosis was inversely correlated with two activity traits, hinting a behavioural syndrome for thanatosis and activity, with bolder individuals exhibiting shorter thanatosis and higher locomotor activity in contrast with fearful individuals, which display longer thanatosis and poor locomotor activity. No relationships were found between the behavioural traits and body size or sex. Results of the principal component analysis (PCA) suggested personality differences among individuals. Dung beetles provide an impressive variety of ecosystem services. Since the provision of these services may depend on the personalities represented in local populations and communities, studies on the ecology of personality in dung beetles should be encouraged in future research.

## 1. Introduction

In animals, a widely applied proxy for “personality” consists of inter-individual behaviour variations which should be repeatable over time and across ecological contexts [1,2,3]. Accordingly, individual personality can be classified as bold, aggressive, social, exploratory, or active [4,5]. The repeatability gives a standardised estimate of the consistency of an individual behaviour [6,7,8]; thence, repeated measures of behaviour should be provided in personality studies [9]. 

A behavioural syndrome is a set of correlated behaviours expressed either within a given behavioural context or across different contexts [10]. It essentially represents the correlation between individual-mean values across suites of traits and requires repeated measures of data for each behaviour [9]. A population, or a species in general, can exhibit a behavioural syndrome, and each individual in the considered group can show a certain personality related to that behaviour (e.g., bolder versus shyer individuals) [11].

Personality studies have generally focused on vertebrates [12]; however, in recent years, several studies have demonstrated the existence of personality and behavioural syndromes in invertebrates, particularly in insects [5,12,13], and most have focused on locomotor behaviour [12,14,15] and thanatosis [16,17].

Locomotor behaviour, commonly referred to as ‘activity’, is an ecologically relevant behaviour often positively correlated with aggressiveness and boldness [18]. Personality has been ascertained in beetles [12,14,15,18,19,20] by testing a variety of activity-related traits such as the covered distance, amount of movements, or latency to walking.

Thanatosis, also known as ‘death-feigning’ or ‘tonic immobility’, is a commonplace response to external stimuli in insects [16,21,22], and it is usually considered an anti-predator behaviour. It has also been proposed to provide an indirect measure of boldness towards predators, which assumes that bolder individuals stay in thanatosis for a shorter period and instead exhibit more activity (i.e., move more) in the presence of a predator. Thus, activity and thanatosis may represent graded alternative anti-predator strategies: fleeing opposed to hiding [19,23]. In line with this hypothesis, a negative correlation between thanatosis and locomotor behaviour was observed in several studies in which the individuals showing shorter periods of time in thanatosis were also more active and considered, therefore, to be bolder [17,24,25].

Sound emission has been broadly studied in insects [26] since stridulation is the mechanism most frequently used by insects to emit sounds. It involves the repeated contact and rubbing of a mobile scraper (plectrum) against a fixed file-like structure (pars stridens) to create a series of pulse trains within a certain frequency range [27,28,29]. In beetles, the acoustic properties of stridulations emitted in response to functional needs may vary between individuals within the same species, as it is the case for male–female interactions, startle display against predators, intraspecific competition, and communication between mother and offspring [30,31,32,33], suggesting thus personality-related characteristics. When disturbed (by predators, or even entomologists), certain beetles generate so-called distress calls, bursts of tooth-strike pulses with distinct and repeated subunits [29,34,35]. To the best of our knowledge, sound emission has never been analysed as an indicator of personality in insects, despite these characteristics being known as potentially personality-related; for example, individuals may exhibit differential tendencies to stridulate, as well as inter-individual differences in distress call parameters that cannot be immediately explained by differences in the size of the stridulatory apparatus components [29].

Dung beetles (Coleoptera, Scarabaeidae) are a large group, comprising around 6200 species divided among 267 genera, with almost global distribution [36]. These beetles have frequently been used as models for the study of phenotypic plasticity [37,38], male dimorphism [39,40,41] and the provision of ecosystem services [42,43,44,45]. Their behaviour has been studied almost exclusively within the framework of reproduction activities, such as the nest-staying behaviour of males [46,47], male–male combat [48,49], the tactics adopted by smaller males to avoid fights with larger ones [50] and parental care displayed by females towards their brood-balls [31,32,51,52].

**Figure 1:**
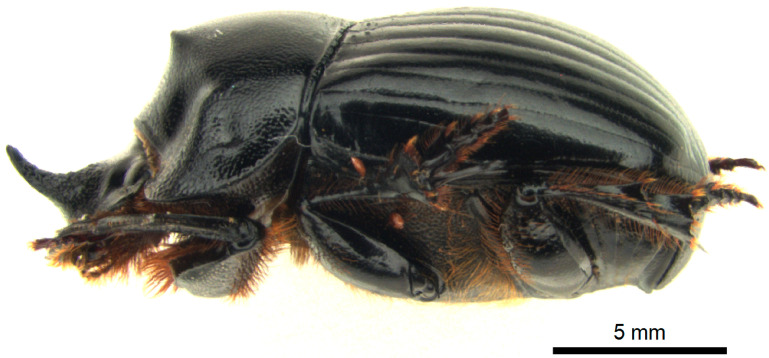
*Copris umbilicatus*, male side view.

In keeping with former studies [53], we examined dung beetle personality and behavioural syndromes to investigate the case of *Copris umbilicatus* Abeille de Perrin, 1901 (Figure 1), which is an optimal model for behavioural research because species of this genus exhibit complex behaviour in a sub-social context [31,32,47]. This coprophagous species is a medium-sized (15–22 mm) tunneller dung beetle with a discontinuous distribution in southeastern Europe [54,55]. It is typical of mid-altitude locations, where it can be found from spring to autumn [56,57] in association with sheep and horse dung, cattle, and even human excrement [57,58].

The aims of our research were threefold, namely, to investigate the potential existence of: (i) personality, by assessing the repeatability of different behaviours over time [59]; (ii) behavioural syndromes, by assessing significant correlations between traits of different behaviours; and (iii) inter-individual differences, by testing for the effect of non-behavioural traits (i.e., sex and/or body size) on the behaviours analysed. We considered three behaviours consisting of seven behavioural traits (i.e., three activity-, one thanatosis- and three distress call-related traits).

## 2. Materials and Methods

### 2.1. Sampling Methods

Fifteen females and ten males of *Copris umbilicatus* were collected by hand in the spring of 2021 from pastures belonging to the Spedaletto Farm in Cantagallo, Tuscany, Italy, (43°59′52.95″ N; 11°01′08.52″ E). Dung beetles were reared kept in the vivarium facilities of the Department of Life Sciences and Systems Biology (DBIOS) at the University of Turin. All specimens were housed in separate plastic terrariums (plastic buckets measuring 20 × 20 cm, diameter × height), at constant temperature (23 °C), from May through to the end of the experiment (June). A unique distinctive alphanumeric code was assigned to each individual to create the database of measures.

### 2.2. Behavioural Assays

To measure the repeatability of a behaviour over time in response to a stimulus, subjects should be tested several times, with short time intervals between each test [1,6]. On the other hand, testing an individual too many times would increase the risk of habituation, which might cause a decrease in responsiveness [6]. As an acceptable trade-off, we decided to carry out two replications of each behavioural assay. Each trial was followed by a period of rest during which beetles were left in their terraria with ad libitum fresh dung. The second trial was performed one week after the first when testing for activity and thanatosis and two weeks later when testing distress calls. All behavioural trials were carried out between 11.00 a.m. and 1.00 p.m. to limit possible interference of the circadian rhythm on beetle activity.

#### 2.2.1. Activity

We assessed the locomotor activity of individuals using an annulus-shaped arena in accordance with previous studies [12,14]. All beetles were given two 3-min runs in the arena, which consisted of two Petri dishes fixed together in such a way as to leave an annulus (a circular runway of diameter 20 mm) in which the beetle could move freely. We experimentally assessed the optimal annulus width (i.e., 20 mm) to make sure it was well suited to *C. umbilicatus*. The arena was divided into eight radial sectors of equal length by drawing lines on the back of the larger Petri dish which intersected at its centre.

We measured the following three behavioural traits: (1) ‘distance moved’, quantified as the total number of radial sectors traversed in a single run; (2) ‘locomotory speed’, calculated as the distance travelled per unit time (mm/s) to cross four radial sectors; and (3) ‘movement duration’, calculated as the overall time (expressed in seconds) spent moving during the run, i.e., the moments of immobility were deducted from the 3-min run. Some individuals reversed the direction of travel during the run. We continued counting the number of sectors and measuring the other behavioural traits as they travelled in the opposite direction.

At the end of each run, before positioning the next individual, the arena was cleaned to remove droppings or possible pheromonal trails on the route, which could influence the activity of the next individual [14]. Each run was video recorded using a camera positioned above the arena so that movements could be accurately measured a posteriori by direct examination.

#### 2.2.2. Thanatosis

We measured thanatosis using a single trait: the time taken for each individual to emerge from stasis after a disturbance—the act of being picked up by hand (thus removed from the soil) and placed in a Petri dish in a supine position. We considered any clear signs of movement, such as attempts to roll over to return to the prone position, as the end of the death-feigning behaviour. If a beetle did not fall into thanatosis, the duration of death-feigning was recorded as zero. 

#### 2.2.3. Distress Signals

Individuals were positioned 1 cm away from a sound level meter model 2235) (Brüel & Kjær, Copenhagen, Denmark) whilst gently holding them by the front legs using two fingers to allow free movement of the abdomen. The sound level meter was calibrated with a 1000 Hz sound produced by a Brüel & Kjær 4230 acoustic calibrator. The sampling rate of the sound recording instrument was set to 48 kHz/16 bit, as it is suitable for detecting stridulations in *Copris lunaris* [29]. Each recording lasted 90 s and contained a varying number of stridulations.

We used the sound analysis software Avisoft-SAS Lab Pro v5.2.13 (2019) (Avisoft Bioacoustics e.K. Glienicke/Nordbahn, Germany) to identify and categorise the sound recordings. All emitted sounds used in our analysis were quantifiable as acoustic distress signals [34,35], consisting of pulse trains with high repetition rates (Figure S1, Table S1).

Using the ‘seewave’ package [60] of the R software v4.2.1 [61], we assessed three traits for each recording. (1) The ‘median amplitude envelope’ quantifies the amplitude change of a sound over time and distinguishes each sound as unique to all others [62,63]. The median amplitude envelope was measured by considering the entire oscillogram for each recording using the *M* function. A recording with an oscillogram containing only a few stridulations will have a value close to 0, whereas a recording with an oscillogram characterised by many stridulations will have a value close to 1. (2) ‘Frequency’ is calculated with the function meanspec as the ratio of the mean frequency to the frequency at the 97.5 quantile of all stridulations in the recording. The frequency at the 97.5 quantile was considered a good approximation of the maximum frequency, which we decided not to use to avoid problems of incorporating sounds not emitted by the insect, namely occasional accidental sounds such as those caused by the operator’s finger or an insect’s leg hitting the sound level meter. (3) ‘Spectral flatness’ is defined as the ratio of the geometric mean to the arithmetic mean of a power spectrum [64,65]. Spectral flatness, calculated with the function sfm considering the entire spectrogram for each recording, quantifies how close a sound is to being a pure tone (i.e., a sound with a sine wave) versus a noise [66]. We can distinguish between a power spectrum, i.e., a classical sound with several peaks, and a flat spectrum, i.e., a sound with a single, continuous peak representing white noise. The value varies from 0 to 1, where 0 represents a pure tone (low spectral flatness) and 1 represents white noise (high spectral flatness) [67]. Individuals emitting sounds with a high spectral flatness value may be defined as the poorer stridulators (a flat spectrogram is obtained when there are few stridulations in the recording).

In addition, we considered another complementary parameter that may indicate individual personality in sound emission, namely the tendency to stridulate. This parameter was calculated by considering the number of recordings which were needed to obtain at least 25 complete stridulations (i.e., stridulations constituted by the subunits a and b; see Figure S1). For some individuals, two recordings were enough as they were excellent stridulators (i.e., they immediately showed a high tendency to stridulate and emit many sounds), whereas up to five recordings were needed from other individuals, which we can describe as being more reluctant to stridulate. Each animal was then assigned a score indicating the tendency to stridulate (0–3, where 0 = poor stridulator and 3 = excellent stridulator).

### 2.3. Body Size Evaluation

The maximum pronotum width provides a reliable approximation of dung beetle body size [68]. Images of the pronotum were thus captured using LAS-Leica Application Suite software (Leica Microsystems AG, Wetzlar, Germany) and a Leica^®^ DMC4500 digital camera connected to a stereoscopic dissecting scope Leica^®^ Z16APO. Morphological data acquisition and measurement were performed according to standard methods previously described [69].

### 2.4. Statistical Analyses

First, we assessed for the repeatability of behaviours (behavioural consistency) to define personality. We then looked for correlations between behaviours to identify behavioural syndromes. Finally, to analyse behavioural differences among individuals, we tested for the contemporaneous effect of non-behavioural traits (i.e., sex and/or body size).

We assessed behavioural consistency (i.e., personality) by quantifying the repeatability coefficient (which can assume a value between 0 and 1) for the seven behavioural traits (i.e., three activity-, one thanatosis- and three bioacoustics-related traits) using the intraclass correlation coefficient (ICC), defining moderate to excellent levels of repeatability (ICC values interpretation based on [70]) between the two trials for all behavioural traits. We calculated the ICC(1,k) [70,71] to assess the mean response repeatability of each trait as the difference between the between-group mean square and the within-group mean square divided by the between-group mean square. We transformed our metrics to satisfy model assumptions (square-root transformation for death feigning duration and median amplitude envelope and logarithmic transformation for distance moved and spectral flatness). After checking the repeatability, we used the mean value for the two trials conducted for each variable for all subsequent tests. We calculated ICC with the function *ICC* of the R package ‘psych’ [72]. Given that the *ICC* function relies on a mixed effect model, we checked model assumption by fitting the same model by using the lmer function of the ‘lme4’ package [73].

To investigate the presence of associations between the different behavioural traits, we calculated Spearman’s rank correlation coefficients. Correlation analyses were conducted in two stages: the first between the traits related to the same behaviour and the second between traits related to different behaviours.

The relationships between each behavioural trait and body size, sex and their interactions were tested by linear regression.

Principal Component Analysis (PCA) was performed on activity and distress call behaviours (three traits for each) separately to look for any trends in the data (e.g., clusters of individuals). PCA was also performed on all three behaviours combined (i.e., all seven traits). Vectors and factor averages were fitted on the PCA results and tested for significance using permutation tests to search for a relationship between the first two PCA coordinates, body size and sex. Furthermore, the squared correlation coefficient (R^2^), representing the proportion of variance explained by these two variables, was calculated to evaluate the relative importance of body size and traits in explaining the PCA results. The PCA was calculated with the rda function of the R package ‘vegan’ [74]. Data were managed and plot generated with the collection of R packages ‘tidyverse’ [75].

## 3. Results

### 3.1. Repeatability

Repeatability estimates (Table 1) were noticeably higher and highly significant in the behavioural traits related to sound emission, modest in traits related to locomotory activity and lower in the trait related to thanatosis. Movement duration and death-feigning duration showed the weakest significance values.

### 3.2. Correlations between Behavioural Traits

Concerning the correlations between traits pertaining to the same behaviour, in relation to ‘activity’, we detected strong positive and statistically significant correlations between distance moved and locomotory speed (ρ = 0.87; *p* < 0.001), between distance moved and movement duration (ρ = 0.71; *p* < 0.001) and between locomotor speed and movement duration (ρ = 0.62; *p* < 0.001); in relation to ‘sound emission’, we found negative and statistically significant correlations between the median amplitude envelope and spectral flatness (ρ = −0.78; *p* < 0.001) and between spectral flatness and frequency (ρ = −0.64; *p* < 0.001). The correlation between median amplitude envelope and frequency was not significant (ρ = 0.28; *p* = 0.181). The tendency to stridulate correlated positively with the median amplitude envelope (ρ = 0.60; *p* < 0.01) and frequency (ρ = 0.45; *p* < 0.05), whereas it correlated negatively with spectral flatness (ρ = –0.75; *p* < 0.001).

With regard to correlations between traits pertaining to the different behaviours, thanatosis duration was negatively correlated with distance moved (ρ = −0. 56; *p* < 0.01) and locomotory speed (ρ = −0.52; *p* < 0.01), which thus suggested the existence of a syndrome. No other significant correlations were found between behavioural traits belonging to different behaviours: there were no significant correlations between sound-related traits and activity, and there were no significant correlations between thanatosis duration and sound-related traits.

### 3.3. Behavioural Differences between Individuals

To investigate the possibility that behavioural differences between individuals might depend on sex and/or body size, we tested the relationship between each behavioural trait and body size, sex and their interactions by means of linear regressions. No relationship was found between the investigated traits and body size, sex and their interactions (Figures S2–S4), except for the frequency stridulation sounds which showed a significant interaction term (F_1,21_ = 5.73, *p*-value < 0.05).

**Figure 2:**
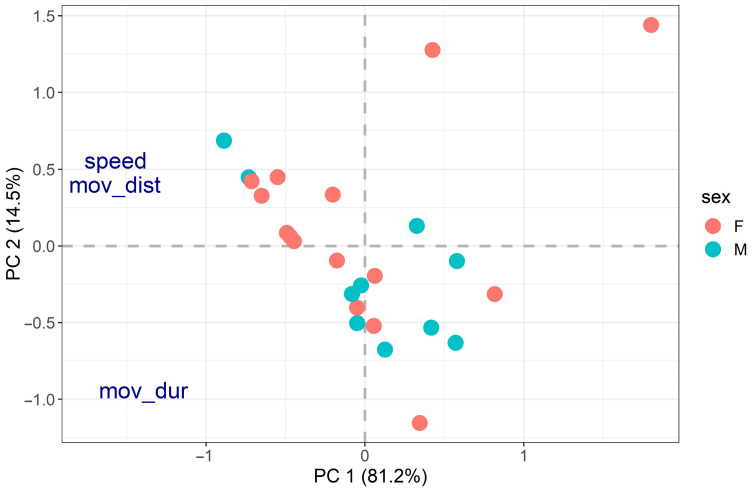
PCA on activity traits (speed = locomotory speed, mov_dist = distance moved, mov_dur = movement duration). The two axes explained 81.2% and 14.5% of the data variability for a total of 95.7%. Distance moved, locomotory speed and movement duration were all negatively related to PC1 (loadings of −1.57, −1.57 and −1.39, respectively). Distance moved (0.40) and locomotory speed (0.43) were positively related to PC2, whereas movement duration (−0.94) was negatively related to PC2. PCA did not reveal any clear behavioural groups, and no significant relationship was found between the PCA results and body size (R^2^ = 7.05%, *p*-value = 0.442) or sex (R^2^ = 3.06%, *p*-value = 0.527).

**Figure 3:**
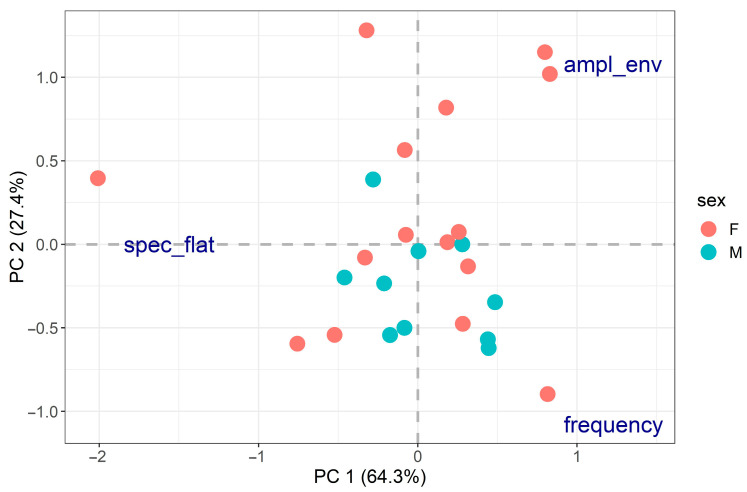
PCA on distress calls (spec_flat = spectral flatness, ampl_env = median amplitude envelope). The two axes explained 64.3% and 27.4% of the data variability for a total of 91.7%. Spectral flatness was mainly related to PC1 (PC1 loading = −1.56, PC2 loading = 0.002), while frequency (PC1 loading = 1.23, PC2 loading = −1.08) and median amplitude envelope (PC1 loading = 1.23, PC2 loading = 1.08) were related to both PC axes. PCA did not reveal any clear behavioural groups, and no significant relationship was found between the PCA results and body size (R^2^ = 10.8%, *p*-value = 0.286) or sex (R^2^ = 7.14%, *p*-value = 0.178).

We then focused on behavioural differences between individuals, considering one behaviour at a time. PCA analyses performed on the separate activity and distress call traits did not reveal any clear behavioural groups (suggesting the presence of great behavioural variation between individuals), and no significant relationship was found between PCA results and body size or sex (Figure 2 and Figure 3).

**Figure 4:**
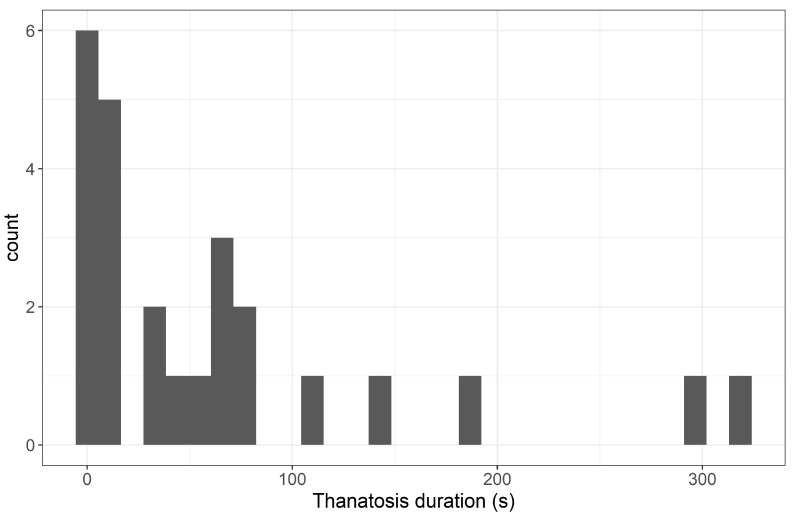
Frequency histogram of thanatosis duration (mean of the two trials) for the 25 individuals tested. Count = number of individuals.

Duration of thanatosis varied from individual to individual. The distribution was right-skewed, with 56% of the individuals showing values below 50 s (Figure 4).

Finally, we tested for behavioural differences between individuals by considering all three multiple behaviours (i.e., all seven behavioural traits) together.

**Figure 5:**
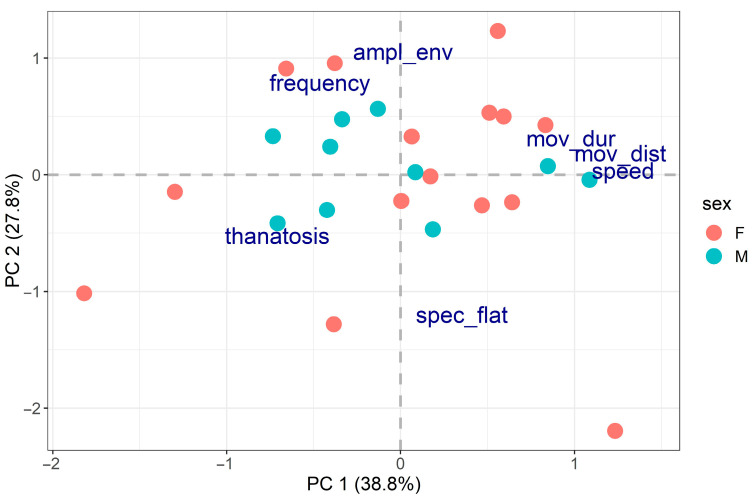
PCA on all multiple behaviours (spec_flat = spectral flatness, ampl_env = median amplitude envelope, speed = locomotory speed, mov_dist = distance moved, mov_dur = movement duration).

In this all-inclusive analysis, the first two PCA axes accounted for 38.8% and 27.8% of the variability in the data for a total of 66.6% (Figure 5).

The first PCA axis was positively related to activity (locomotory speed, distance moved and movement duration) and negatively related to thanatosis (Table 2). This result is consistent with the negative correlation between the two behaviours we found in the previous analyses. The second axis was related to distress calls and showed a negative relationship with spectral flatness and a positive relationship with amplitude envelope and frequency (Table 2). Individuals were well scattered across the PCA bi-dimensional space, suggesting that when all behaviours are considered together, each individual displays a behavioural mix that distinguishes it from all the others. Once again, no significant relationships were found between the PCA results and body size (R^2^ = 5.20%, *p*-value = 0.569) or sex (R^2^ = 0.33%, *p*-value = 0.938), providing strong evidence that these two parameters are not responsible for the inter-individual behavioural differences found.

## 4. Discussion

The present study clearly revealed behavioural differences among individuals, such that the mix of behavioural traits displayed by any one individual distinguished it from all others, contextually showing that neither sex nor body size could account for these differences.

### 4.1. Evidence of Personality

All the behavioural traits measured were repeatable, confirming the consistency of behaviour over time. The present finding suggested that the dung beetle *Copris umbilicatus* expresses personality, keeping with what has been found in other studies on personality related to locomotory activity in *Callosobruchus maculatus* [20], *Tribolium castaneum* [18] and *Nebria brevicollis* [12], and thanatosis in *Tribolium confusum* [24], *Phaedon cochleariae* [19], *Tenebrio molitor* [76], *Onthophagus furcatus* and *O. ruficapillus* [53].

The high repeatability values of the behavioural traits related to the emission of distress calls are utmost interesting, since sound emissions are commonly regarded as a consequence of stress or intraspecific communication rather than a possible personality-defining trait. Although it has been suggested that certain sound parameters may depend on the morphology of the stridulatory organs [27,28], the tendency to stridulate can be nevertheless considered a behavioural parameter independent from morphology. Since here we demonstrated that the tendency to stridulate is significantly correlated with the three selected acoustic parameters, we can be reasonably confident that their high repeatability does not depend on the features of the stridulatory organs.

### 4.2. Correlations between Traits and Evidence of Behavioural Syndromes

Significant correlations were obtained between traits belonging to the same behaviour, with high levels of correlation between traits related to distress signals as well as between traits related to locomotor activity. The presence of intra-behavioural correlations suggests that these traits must be considered together because they contribute to describe behaviours as locomotory activity and distress calls as formerly defined [12,15,19].

We also highlighted a significant correlation between traits belonging to different behaviours, namely thanatosis duration and two activity parameters. Likely, individuals feigning death for a longer timespan were also those which run for shorter distances and at lower locomotory speed; the opposite was also true as those feigning death for a shorter timespan tended to run longer distances at a higher locomotory speed. These results suggest the existence of a behavioural syndrome linking thanatosis and activity as previously detected in mustard leaf beetles [19], ground beetles [12] and rove beetles [15]. Thanatosis may be considered as an indicator of an individual level of boldness, i.e., the tendency of an individual to adopt risk-prone or risk-averse behaviours [10,16,19,77]. According to the findings about *C. umbilicatus*, it is foreseeable that a bold individual exhibits shorter thanatosis and higher locomotor activity, whereas a fearful individual displays longer thanatosis and poor locomotor activity.

Thanatosis and locomotor activity can be considered alternative and opposite anti-predatory strategies [16,24,25]. As previous studies have shown, the anti-predator behaviour exhibited by an individual may be influenced by the presence/absence of predators at the collection site and by the type of predator they most commonly come up against [17,78], as the predator itself may modify the prey’s responses; for example, prey are more likely to develop strong anti-predator behaviour (e.g., longer thanatosis) in a site where the predator is highly present [17]. Typical predators of dung beetles are medium-sized carnivores such as foxes and corvids [79]. Both corvids and mammal predators such as foxes are apt at catching fast-moving prey, but they will also feed on carrion, suggesting that they would not hesitate in pursuing running insects or feed on insects feigning death. Moreover, the activity behaviour we monitored in *C. umbilicatus* may be related to exploratory behaviour, the latter one being directed at acquiring information about the environment [80]. This interpretation is sustained by our observation that when many individuals stopped walking, they nevertheless continued to move their antennas, while others changed their walking direction and even retraced their steps, which is a choice decidedly inconsistent with a fleeing or escape behaviour.

Stridulations emitted by *Copris umbilicatus* individuals were not correlated with either thanatosis or activity. In many insects, stridulations have been identified to be an anti-predatory strategy [26,81,82]. However, certain corvid species (namely the jay, *Garrulus glandarius*) may use stridulations to locate and catch their prey, such as cicadas, *Cicada orni* [83]. It should also be mentioned that in another *Copris* species, namely, *Copris lunaris*, females were seen to emit stridulations to repel experimentally introduced unfamiliar conspecifics away from their nesting areas [31,84] and, in the same way, females emitted excited sound emission when their cocoons were removed by the experimenter [29]. In short, stridulations in dung beetles may be more closely associated with offspring-defensive behaviour than with anti-predatory behaviour.

All of the above considered, further ad hoc studies are needed to ascertain and understand the possible ecological implications of thanatosis, activity and stridulations in dung beetles.

### 4.3. Different Individuals Display Different Behaviours

We had expected that behavioural differences among individuals could depend on sex and/or body size; except for stridulation frequency, no relationships between the investigated traits and body size, sex and their interactions were found using linear models. Regarding the frequency of the stridulation sounds emitted by *C. umbilicatus*, a significant, albeit weak, interaction was revealed. Opposite trends were found in the two sexes about the body size/frequency relationship: being male was associated with higher frequencies for larger body sizes, whereas the opposite was true in females, with frequencies decreasing as body size increased. The results of PCA also sustained that the behavioural characteristics do not depend on sex or body size. Discordant results have been obtained in insects, with some studies suggesting that sex does not significantly influence personality [14,19,24], and others revealing different personalities according to sex [85,86] or body size [21].

The most relevant result of the PCA analyses concerns the ordination of individuals, which were highly scattered in the bi-dimensional plane, particularly in the analysis performed on multiple behaviours. The scattered distribution reflects the expression of different behaviours by different individuals; in other words, each individual displays behavioural characteristics that distinguish it from all the others, which is in accordance with the results of several previous studies [3,87,88]. One plausible explanation for this noticeable inter-individual behavioural differentiation, which does not depend on sex or body size, is that it is the manifestation of the different personalities expressed by individuals.

Incidentally (and interestingly), PCA also confirmed and reinforced the results of the correlation analyses: locomotor behaviour and thanatosis were located on the same axis, but the direction of the correlations was opposite (in keeping with the negative correlation between the two behaviours), whereas the traits relating to distress calls were located on different axes (in keeping with the lack of a correlation between the acoustic signal and the other two behaviours).

## 5. Conclusions

Given the importance of dung beetles to ecosystem functionality, the study of their personality could be useful for gaining a better understanding of the ecosystem services that these beetles offer. Through the manipulation of livestock faeces for their feeding and nesting processes, dung beetles contribute, first and foremost, to dung removal, but also to bioturbation, nutrient cycling, mineralization processes, plant nutrient uptake and plant growth enhancement [42,89], all of which may benefit agricultural and pastoral ecosystems. Future research might reveal bolder or more active individuals to be those able to remove more dung. Consequently, a population with many bold and active individuals might be more efficient at providing this primary ecosystem service than a population characterised by shyer and less active individuals. This could obviously be extended to communities with different species of dung beetles. By consequence, we recommend that studies on the ecology of dung beetle personality be encouraged and further research performed.

## Figures and Tables

**Table 1:** Intraclass correlation coefficient (ICC) values for each behavioural trait (* *p* < 0.05, ** *p* < 0.01, *** *p* < 0.001). Statistical tests were performed to test the null hypothesis that ICC differed from 0.

Behaviour	Behavioural Trait	ICC	*p*-Value
Activity	distance moved	0.66	**
	locomotory speed	0.66	**
	movement duration	0.58	*
Thanatosis	death feigning duration	0.56	*
Distress calls	median amplitude envelope	0.90	***
	frequency	0.77	***
	spectral flatness	0.90	***

**Table 2:** Loadings of the first two PCA axes for each behavioural trait.

Behavioural Trait	PC1 Loading	PC2 Loading
Locomotory speed	1.28	0.04
Distance moved	1.26	0.19
Movement duration	0.98	0.23
Spectral flatness	0.35	−1.20
Median amplitude envelope	0.01	1.06
Frequency	−0.46	0.88
Thanatosis	−0.71	−0.43

## Data Availability

The data are available at reasonable request from the corresponding author.

## Supplementary Materials

The following supporting information can be downloaded at: https://www.mdpi.com/article/10.3390/insects14060529/s1, Figure S1: Visualisation of acoustic distress signals [29,34,35]; Figure S2: Relationship between body size and behavioural traits; Figure S3: Boxplots for the two sexes showing the median value, interquartile range and outliers for each behavioural trait tested; Figure S4: Relationship between sex, body size and the behavioural traits tested; Table S1: Summary statistics; Video S1: Activity Pattern.
