# Supplementary material for: Behavioural Repeatability and Behavioural Syndrome in the Dung Beetle Copris umbilicatus (Coleoptera, Scarabaeidae)
Source: Insects. 2023 Jun 6;14(6):529. doi: 10.3390/insects14060529 (PMC10298848; doi:10.3390/insects14060529)
Supplement: Supplementary file 1 [file insects-14-00529-s001.zip › insects-2409337-supplementary.pdf]

Supplementary Material

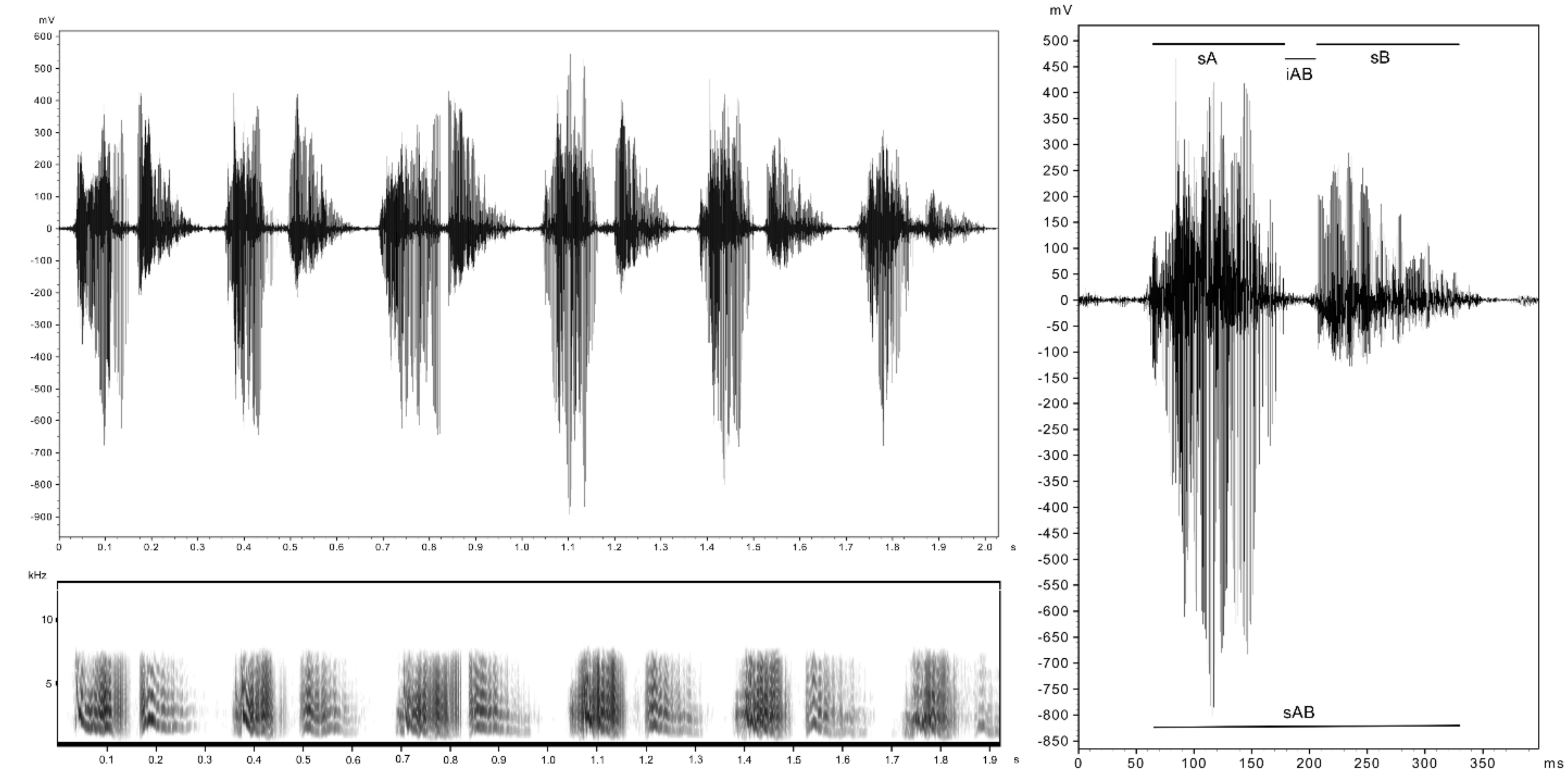

Figure S1. Visualisation of acoustic distress signals. (a) Oscillographic and (b) spectrographic representation of a pulse train with complete stridulations. (c) Each complete stridulation (sAB) is composed of two subunits of opposite phases with harmonic structure: subunit A (sA) is emitted during the extension of the abdomen, whereas subunit B (sB) is emitted when the abdomen has contracted back to its original position. The two subunits are separated by a short interval (iAB). The distress calls emitted by C. umbilicatus share the same characteristics already described for Copris lunaris, C. hispanus and C. incertus [29,34,35].

Table S1. Summary statistics. Mean and standard deviation of the acoustic parameters of males (12 individuals, number of complete stridulations N = 300) and females (14 individuals, N = 350). We captured two temporal and four spectral parameters: (i) temporal length of subunit/pulse A (dA); (ii) temporal length of subunit/pulse B (dB); (iii) peak frequency at the maximum parameter of subunit/pulse A (PF_A); (iv) peak frequency at the maximum parameter of subunit/pulse B (PF_B); (v) fundamental frequency at the maximum parameter of subunit/pulse A (FF_A); (vi) fundamental frequency at the maximum parameter of subunit/pulse B (FF_B). Data were reported in the following units of measurement: dA and dB in milliseconds; PF_A, PF_B, FF_A and FF_B in Hertz. These acoustic parameters were selected in order to compare the distress calls emitted by *Copris umbilicatus* with those previously analysed in other species of the genus *Copris*.

	Males	Females
dA	77.447 ± 14.611	91.142 ± 18.177
dB	103.340 ± 13.601	111.398 ± 10.373
PF_A	3268.900 ± 474.584	3103.600 ± 364.997
PF_B	2928.733 ± 280.072	2719.286 ± 309.043
FF_A	1769.667 ± 271.950	1515.943 ± 283.431
FF_B	1555.833 ± 444.734	1467.257 ± 378.140

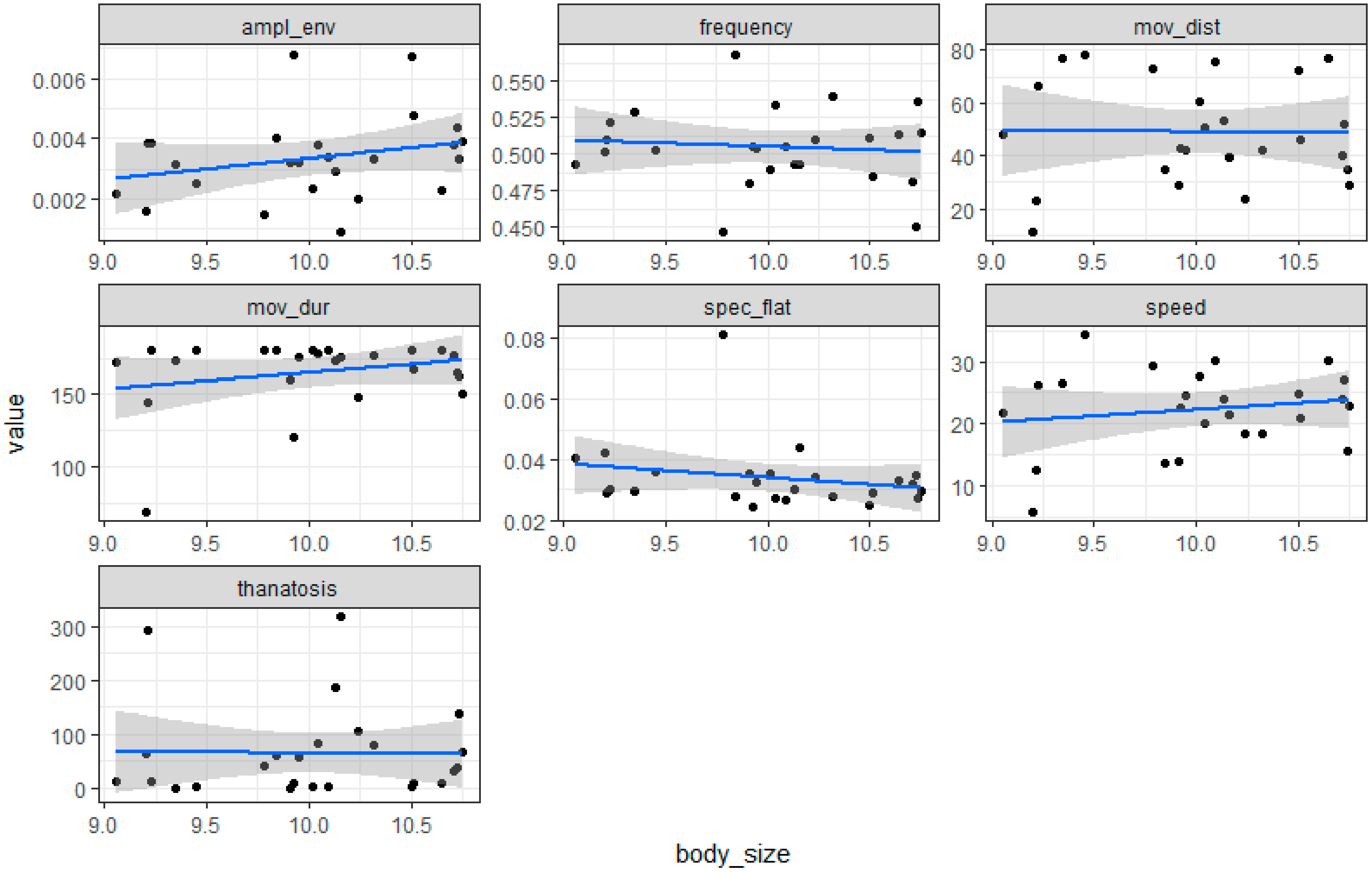

Figure S2. Relationship between body size (x axis) and the behavioural traits tested (y axis; ampl_env = median amplitude envelope, frequency, mov_dist = distance moved, mov_dur = movement duration, spec_flat = spectral flatness, speed = locomotory speed, thanatosis).

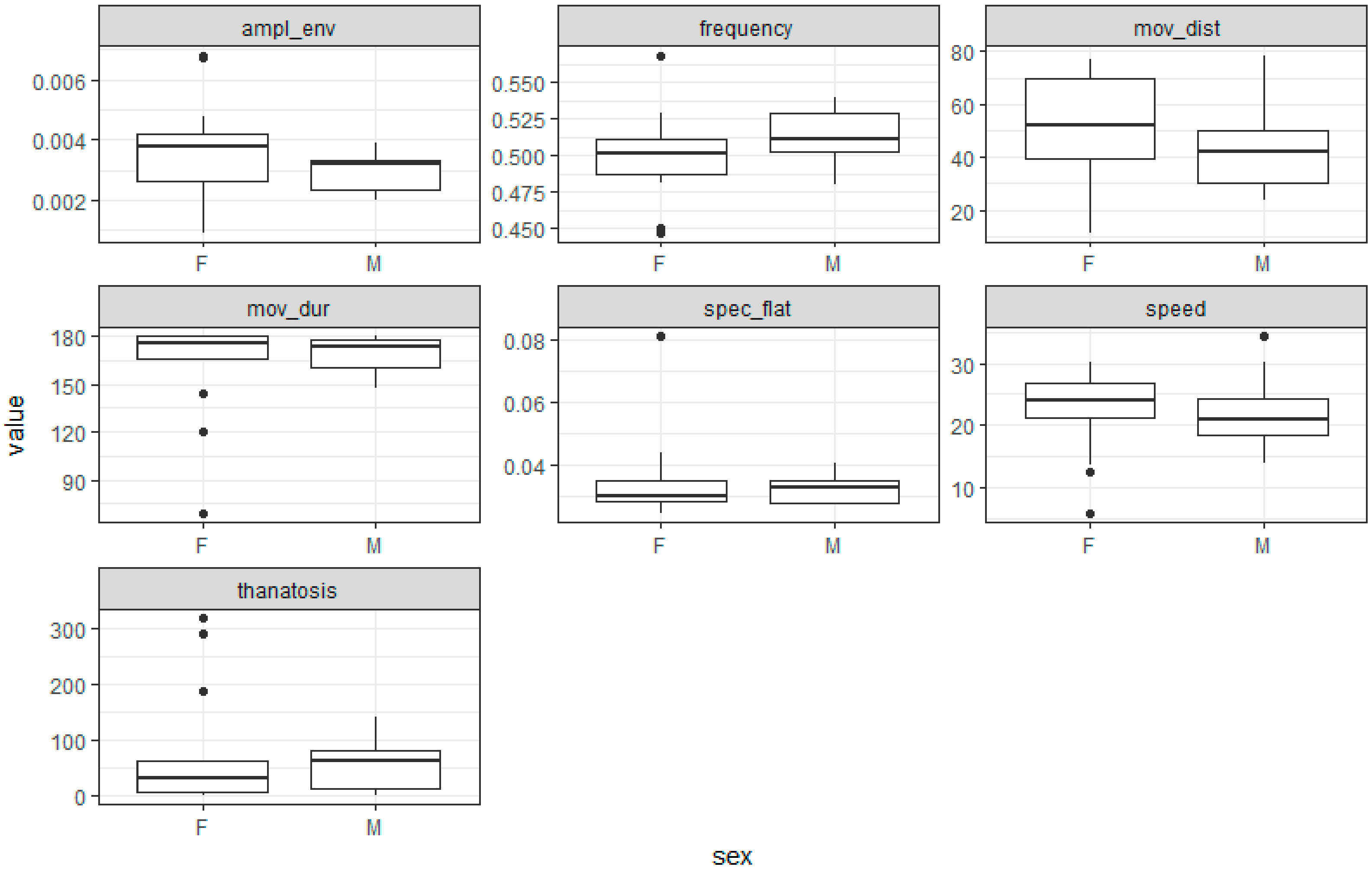

Figure S3. Boxplots for the two sexes (x axis) showing the median value, interquartile range and outliers for each behavioural trait tested (y axis; ampl_env = median amplitude envelope, frequency, mov_dist = distance moved, mov_dur = movement duration, spec_flat = spectral flatness, speed = locomotory speed, thanatosis). No differences were found between the two sexes in any of the behavioural traits.

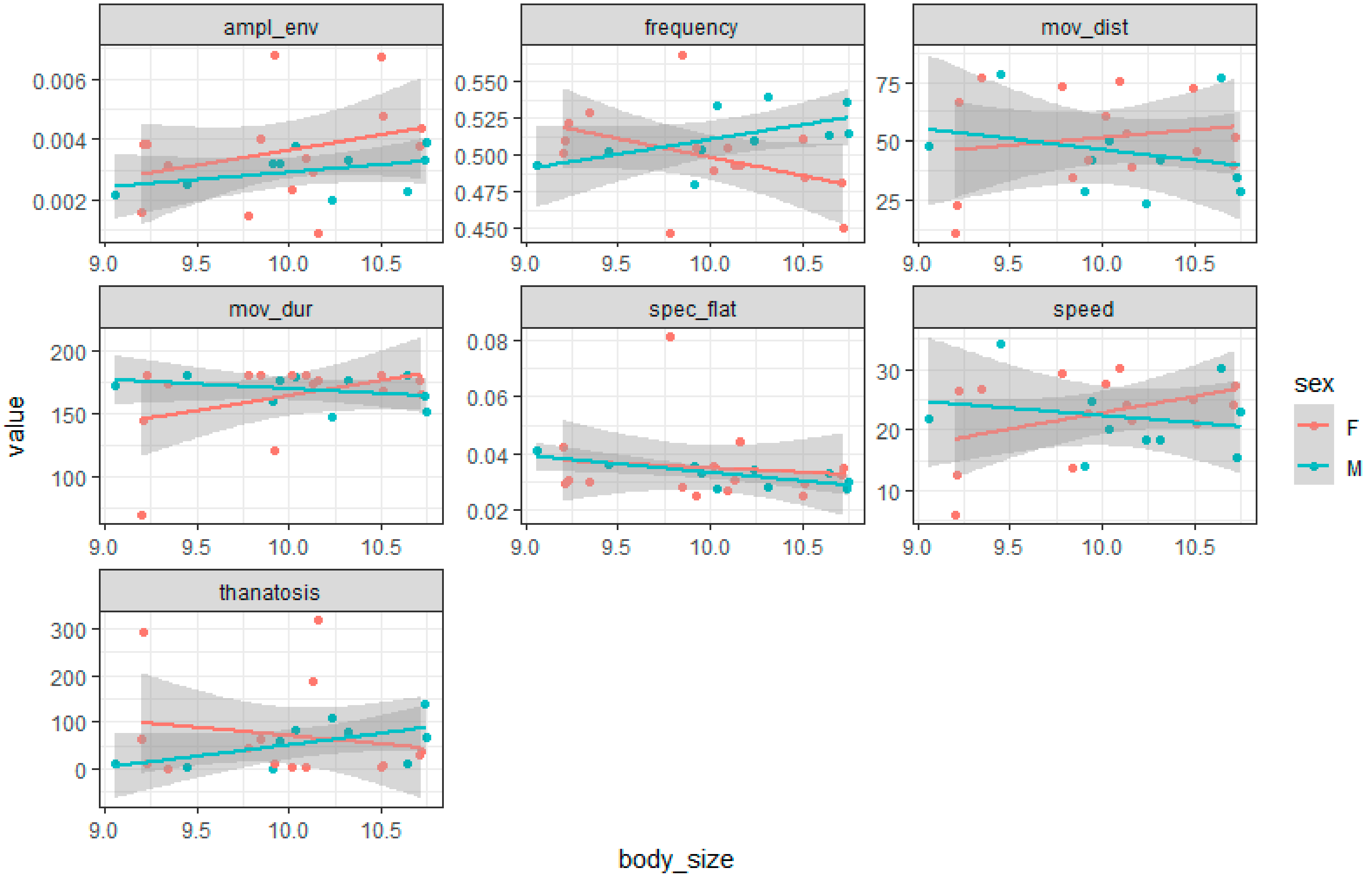

Figure S4. Relationship between sex, body size and the behavioural traits tested (ampl_env = median amplitude envelope, frequency, mov_dist = distance moved, mov_dur = movement duration, spec_flat = spectral flatness, speed = locomotory speed, thanatosis). A regression line and its confidence interval were drawn for each sex and behavioural trait. Interaction between body size and sex was not significant for all the behavioural traits tested, except for frequency ($F_{1,21}$ = 5.73, p-value < 0.05).

1. Kerman, K.; Roggero, A.; Piccini, I.; Rolando, A.; Palestrini, C. Dung Beetle Distress Signals May Be Correlated with Sex and Male Morph: A Case Study on *Copris lunaris* (Coleoptera: Scarabaeidae, Coprini). *Bioacoustics* **2021**, *30*, 180–196. https://doi.org/10.1080/09524622.2019.1710255.
2. Palestrini, C.; Pavan, G.; Zunino, M. Acoustic Signals in *Copris incertus* Say (Coleoptera Scarabaeidae Coprinae). *Ethol. Ecol. Evol.* **1991**, *3*, 143–146. https://doi.org/10.1080/03949370.1991.10721928.
3. Palestrini, C.; Pensati, F.; Barbero, E.; Laiolo, P. Distress Signals in *Copris hispanus* (L., 1764) (Coleoptera: Scarabaeidae). *Insect Soc. Life* **2000**, *3*, 185–193.
